# Transcriptomic Reprogramming and Key Molecular Pathways Underlying Huanglongbing Tolerance and Susceptibility in Six Citrus Cultivars

**DOI:** 10.3390/ijms26157359

**Published:** 2025-07-30

**Authors:** Xiaohong Chen, Fang Fang, Tingting Chen, Jinghua Wu, Zheng Zheng, Xiaoling Deng

**Affiliations:** Guangdong Province Key Laboratory of Microbial Signals and Disease Control, South China Agricultural University, Guangzhou 510642, China; 13211213504@163.com (X.C.); m18307922033@163.com (F.F.); 15213724782@139.com (T.C.); 13909709515@163.com (J.W.)

**Keywords:** huanglongbing, citrus cultivar, tolerant, susceptible, host response

## Abstract

Huanglongbing (HLB), caused by *Candidatus* Liberibacter asiaticus (CLas), is the most devastating disease threatening global citrus production. Although no commercial citrus varieties exhibit complete HLB resistance, genotype-specific tolerance variations remain underexplored. This study conducted a comparative transcriptomic profiling of six commercially citrus cultivars in South China, four susceptible cultivars (*C. reticulata* cv. Tankan, Gongkan, Shatangju, and *C. sinensis* Osbeck cv. Newhall), and two tolerant cultivars (*C. limon* cv. Eureka; *C. maxima* cv Guanxi Yu) to dissect molecular mechanisms underlying HLB responses. Comparative transcriptomic analyses revealed extensive transcriptional reprogramming, with tolerant cultivars exhibiting fewer differentially expressed genes (DEGs) and targeted defense activation compared to susceptible genotypes. The key findings highlighted the genotype-specific regulation of starch metabolism, where β-amylase 3 (*BAM3*) was uniquely upregulated in tolerant varieties, potentially mitigating starch accumulation. Immune signaling diverged significantly: tolerant cultivars activated pattern-triggered immunity (PTI) via receptor-like kinases (*FLS2*) and suppressed ROS-associated RBOH genes, while susceptible genotypes showed the hyperactivation of ethylene signaling and oxidative stress pathways. Cell wall remodeling in susceptible cultivars involved upregulated xyloglucan endotransglucosylases (*XTH*), contrasting with pectin methylesterase induction in tolerant Eureka lemon for structural reinforcement. Phytohormonal dynamics revealed SA-mediated defense and NPR3/4 suppression in Eureka lemon, whereas susceptible cultivars prioritized ethylene/JA pathways. These findings delineate genotype-specific strategies in citrus–CLas interactions, identifying *BAM3*, *FLS2*, and cell wall modifiers as critical targets for breeding HLB-resistant cultivars through molecular-assisted selection. This study provides a foundational framework for understanding host–pathogen dynamics and advancing citrus immunity engineering.

## 1. Introduction

Citrus Huanglongbing (HLB), a devastating global disease threatening citrus production for over a century, systematically infects trees across all developmental stages. Typical HLB symptoms included chlorotic shoots, mottled leaves, fruit deformation, and premature abscission, culminating in rapid tree decline and mortality [1,2]. HLB is caused by the unculturable phloem-limited α-proteobacterium, comprising three species, i.e., *Candidatus* Liberibacter asiaticus (CLas), *Ca*. L. africanus (CLaf), and *Ca*. L. americanus (CLam). CLas has dominated Chinese outbreaks since its initial identification in Guangdong Province [3,4]. Currently, no cure is available for HLB. Although most commercial citrus cultivars exhibited susceptibility to HLB, certain genotypes demonstrated natural tolerance to CLas infection [5]. Notably, HLB tolerance was observed in the species *Citrus limon* (lemon) and *C. maxima* (pomelo), whereas *C. sinensis* (sweet orange) and *C. reticulata* Blanco (mandarin) exhibited susceptibility to the pathogen [6]. These findings were consistent with earlier reports suggesting genotype-dependent tolerance mechanisms in citrus [5,7]. In addition, studies also identified pivotal HLB-resistant citrus genes, including *CitPH4* (MYB transcription factor) [8], *CsHIPP03* (heavy metal-associated isoprenylated protein) [9], and *ClDJC24* (co-chaperone) [10] and *MYB2* [11]. These discoveries advanced molecular breeding strategies to enhance citrus immunity against CLas pathogenesis.

RNA sequencing (RNA-seq) has emerged as a pivotal tool in elucidating the molecular mechanisms underlying citrus–CLas interactions, providing critical insights into citrus–CLas dynamics and cultivar-specific responses. Current studies demonstrate that CLas induced extensive metabolic reprogramming in citrus hosts, characterized by the preferential activation of ATP biosynthesis and glycolytic pathways to sustain bacterial proliferation, concurrent with the systemic suppression of photosynthetic machinery and defense-related signaling cascades [6,12]. Seasonal transcriptomic profiling revealed distinct temporal regulation of immune responses, with spring emergence exhibiting a pronounced activation of pathogen-associated molecular pattern (PAMP)-triggered immunity components, including NBS-LRR receptors, receptor-like kinases (RLKs), and pathogenesis-related (PR) proteins, thereby reinforcing HLB as an pathogen-triggered immune disease [13,14]. An integrative analysis of citrus transcriptomic datasets also identified conserved transcriptional signatures across diverse citrus genotypes, with leaf-specific transcriptional networks showing particular sensitivity to HLB progression, suggesting tissue-specific vulnerability patterns [15]. These multilayered investigations collectively established a framework for understanding HLB/CLas pathogenesis while highlighting potential targets for precision breeding strategies.

This study present a comprehensive investigation into genotype-specific responses to HLB pathogenesis across six major commercially cultivated citrus varieties in South China, including *Citrus reticulata* cv. Gongkan, *C. reticulata* cv. Tankan, *C. reticulata* Blanco cv. Shatangju, *C. limon* cv Eureka, *C. maxima* cv Guanxi Yu, and *C. sinensis* Osbeck cv. Newhall. Through a systematic comparative analysis of RNA-seq profiles derived from asymptomatic and symptomatic leaf tissues, we elucidated distinct molecular pathways underlying differential susceptibility to CLas infection. The multi-variety experimental design enabled the identification of key molecular components associated with pathogen recognition, immune activation, and metabolic reprogramming that collectively determine host compatibility. Particularly, comparative transcriptomic profiling revealed conserved and divergent regulatory networks between tolerant and susceptible genotypes, highlighting critical targets for resistance engineering. These findings provided a robust framework for understanding host–pathogen dynamics in citrus–CLas interactions and established functional genomic resources for breeding programs aimed at developing CLas-resistant citrus cultivars through molecular-assisted selection.

## 2. Results

### 2.1. Symptoms and CLas Quantification of Leaf Samples from Six HLB-Affected Citrus Cultivars

**Figure 1:**
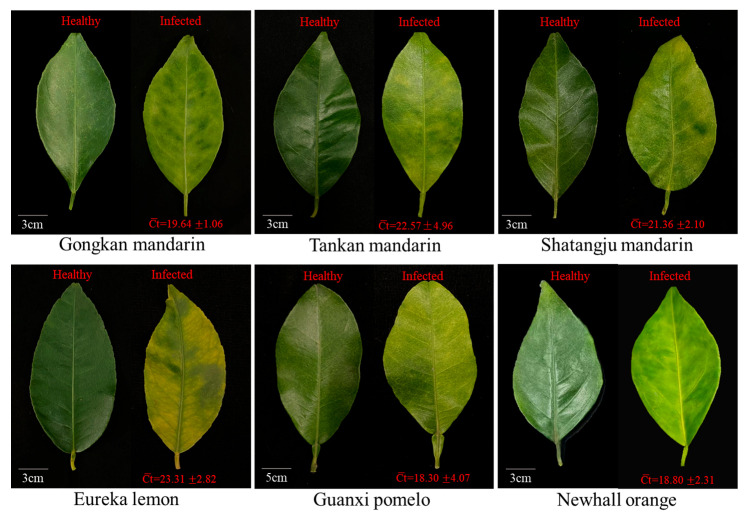
Representative leaf phenotypes and CLas quantification in six citrus cultivars. CLas titers measured by qPCR in leaves (mean ± SD, n = 3).

HLB-affected leaves displayed characteristic symptoms including pronounced yellowing leaves in Tankan mandarin, Newhall orange, Shatangju mandarin, and Gongkan mandarin, while Eureka lemon and Guanxi pomelo showed typical mottling leaves (Figure 1). For each cultivar, asymptomatic leaves were collected from healthy trees and tested by quantitative PCR. None of these healthy controls yielded detectable CLas (Ct > 38 or undetermined), confirming their uninfected status. The quantification of symptomatic leaves revealed significant interspecific variation in CLas titer: Guanxi pomelo harbored the highest CLas concentration (average Ct value of 18.30 ± 4.07), followed by Newhall orange (18.80 ± 2.31), Gongkan mandarin (19.64 ± 1.06), Shatangju mandarin (21.36 ± 2.10), Tankan mandarin (22.57 ± 4.96), and Eureka lemon (23.31 ± 2.82) (Figure 1, Supplementary Table S3).

### 2.2. Identification of DEGs

**Figure 2:**
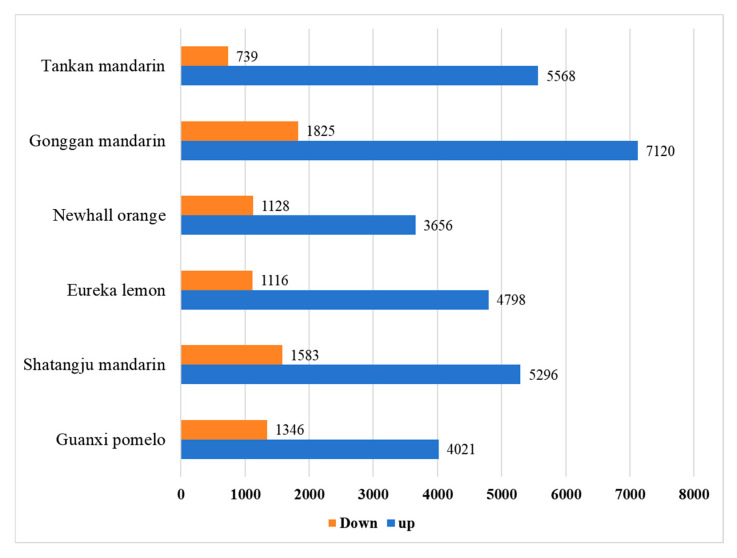
Statistical plot of differentially expressed genes (DEGs) in six citrus varieties in response to *Candidatus* Liberibacter asiaticus infection.

**Figure 3:**
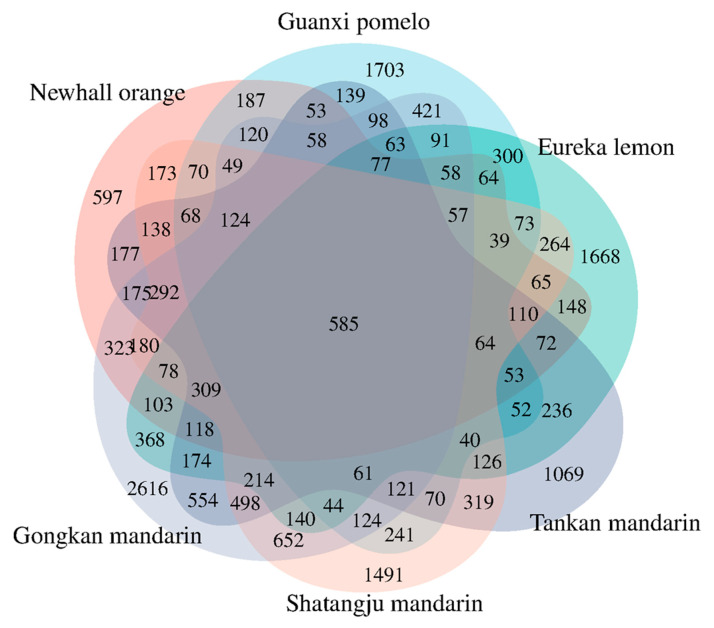
Venn diagram of differentially expressed genes (DEGs) in six varieties in response to *Candidatus* Liberibacter asiaticus infection.

RNA sequencing yielded 36 high-quality libraries, each producing 40–55 million paired-end reads (150 bp; Q30 > 86%) with reference genome alignment rates of 74.09–92.45% (Supplementary Table S1). The biological replicates were highly correlated, with Pearson’s correlation coefficient ranging from 0.612 to 1 (Supplementary Table S2). DESeq2 analysis (|log2FC| ≥ 1.5, *padj* < 0.05) revealed extensive transcriptional reprogramming across citrus cultivars (Figure 2). The number of DEGs varied among citrus cultivars, including 6307 DEGs in Tankan mandarin (5568 up, 739 down), 8945 DEGs in Gongkan mandarin (7120 up, 1825 down), 6879 DEGs in Shatangju mandarin (5296 up, 1583 down), 4784 DEGs in Newhall orange (3656 up, 1128 down), 5914 DEGs in Eureka lemon (4798 up, 1116 down), and 5367 DEGs in Guanxi pomelo (4021 up, 1346 down). Comparative analysis identified 585 conserved DEGs across all cultivars (Figure 3). Cultivar-specific responses were prominent, as seen in Tankan mandarin (1069 DEGs), Gongkan mandarin (2616 DEGs), Newhall orange (597 DEGs), Shatangju mandarin (1491 DEGs), Eureka lemon (1688 DEGs), and Guanxi pomelo (1703 DEGs), suggesting both conserved and cultivar-specific mechanisms underlying HLB adaptation.

### 2.3. Gene Ontology (GO) and KEGG Enrichment Analysis

A GO analysis of DEGs revealed distinct functional enrichment patterns between cultivars. Susceptible cultivars showed 33 (Tankan mandarin), 8 (Gongkan mandarin), 17 (Newhall orange), and 16 (Shatangju mandarin) significantly enriched GO terms, with 6 shared terms mainly including DNA metabolic processes and RNA-directed DNA polymerase activity (Supplementary Table S4). In contrast, tolerant cultivars exhibited 27 (Eureka lemon) and 28 (Guanxi pomelo) enriched terms, sharing 13 functional categories such as carbon fixation, NADH dehydrogenase activity, and ribulose-bisphosphate carboxylase activity (Supplementary Table S4). KEGG pathway analysis identified six conserved significantly enriched pathways across all cultivars: ubiquinone biosynthesis, photosynthesis, glyoxylate metabolism, plant–pathogen interaction, and cysteine/methionine metabolism (Supplementary Table S5).

To further identify the DEGs that are related to the susceptibility and tolerance of citrus in response to HLB, the categories of DEGs related to disease response in the susceptible cultivar group (Tankan mandarin, Newhall orange, Shatangju mandarin, and Gongkan mandarin) and tolerant cultivar group (Eureka lemon and Guanxi pomelo) are described in detail in the following section.

Furthermore, to delineate transcriptional mechanisms underlying HLB susceptibility and tolerance, the categories of DEGs related to disease response in the susceptible cultivar group (Tankan mandarin, Newhall orange, Shatangju mandarin, and Gongkan mandarin) and tolerant cultivar group (Eureka lemon and Guanxi pomelo) are comparatively analyzed in detail in the following section.

### 2.4. Starch and Sucrose Metabolism

**Figure 4:**
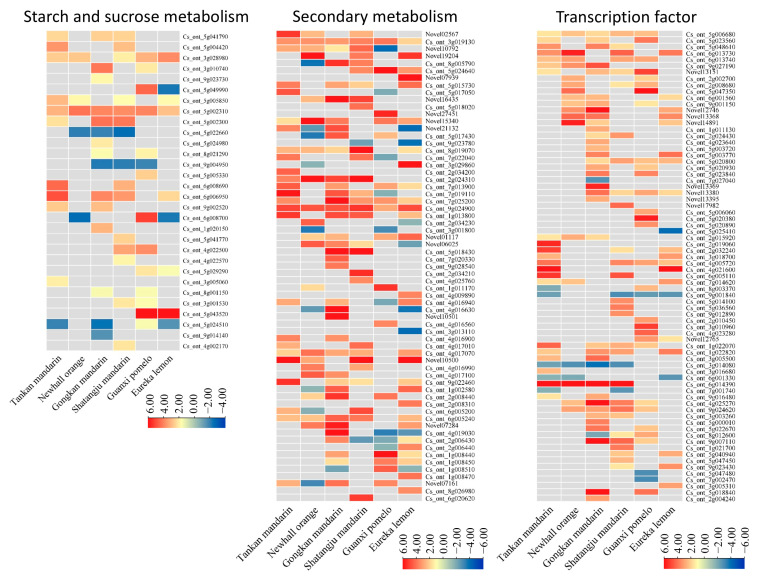
Heatmaps of differentially expressed genes (DEGs) involved in starch and sucrose metabolism, secondary metabolism, and transcription factors.

Transcriptional profiling identified 30 DEGs associated with starch and sucrose metabolism across the six citrus cultivars (Figure 4; Supplementary Table S6). Trehalose metabolism-related genes showed pronounced regulation. Six genes encoding trehalose synthesis/degradation enzymes were universally induced in all cultivars, with the exception of a trehalase gene (*Cs_ont_5g049990*) suppressed in Eureka lemon. Starch synthesis-related genes exhibited divergent expression patterns, with eight genes showing cultivar-specific regulation and minimal differential expression in Eureka lemon. Notably, the putative fructokinase-5 gene (*Cs_ont_5g002310*) demonstrated consistent upregulation across all genotypes. In starch degradation pathways, the β-amylase 3 gene (*Cs_ont_5g043520*) was strongly upregulated in tolerant cultivars (Guanxi pomelo and Eureka lemon) but remained unaltered in four susceptible genotypes. Among sucrose metabolism-related genes, three probable sucrose-phosphate synthase genes (*Cs_ont_5g024510*, *Cs_ont_9g014140*, *Cs_ont_4g002170*) were predominantly downregulated in six cultivars, except for partial upregulation in Shatangju mandarin and Guanxi pomelo. Nine sucrose degradation-related genes were mostly upregulated in all cultivars, with only *Cs_ont_6g008700* showing downregulation in Eureka lemon and Newhall orange.

### 2.5. Secondary Metabolism

A total of 65 DEGs associated with secondary metabolism were identified. These DEGs were mainly involved in flavonoid biosynthesis, phenylpropanoid metabolism, and terpenoid biosynthesis (Figure 4; Supplementary Table S7). Shikimate O-hydroxycinnamoyltransferase (HST) genes displayed broad activation, with three isoforms (*Novel19204*, *Cs_ont_5g024640*, *Novel07939*) exhibiting strong induction in susceptible Newhall, tolerant Guanxi pomelo, and Eureka lemon. Six phytoalexin biosynthesis genes, including *vinorine synthase* and *chalcone synthase*, were markedly upregulated in susceptible cultivars (Tankan mandarin, Gongkan mandarin, and Shatangju mandarin). In contrast, an anthranilate N-methyltransferase gene (*Novel21132*), involved in acridone alkaloid biosynthesis, was downregulated in Newhall orange and strongly suppressed in tolerant Eureka lemon.

Phenylpropanoid metabolism analysis revealed 16 peroxidase-encoding DEGs with divergent regulation among six cultivars. These genes were significantly upregulated in susceptible genotypes but partially downregulated in tolerant varieties. Lignin biosynthesis-related genes, including two *4-coumarate-CoA ligase-like* (*4CL*; *Cs_ont_7g022040*, *Cs_ont_3g029860*), one *cinnamoyl-CoA reductase 1* (*CCR1*; *Cs_ont_1g011170*), and one *caffeoyl-CoA O-methyltransferase* (*CCoAOMT*; *Cs_ont_4g009890*), were highly induced specifically in Eureka lemon and Guanxi pomelo. Terpenoid biosynthesis genes, including *germacrene D synthase* and *β-farnesene synthase*, exhibited genotype-dependent expression, with the maximal induction observed in Gongkan mandarin and Eureka lemon.

### 2.6. Transcription Factors

A total of 74 transcription factor (TF)-associated differentially expressed genes (DEGs) were identified, primarily belonging to the WRKY, MYB, and bHLH families (Figure 4; Supplementary Table S8). WRKY family TFs exhibited pronounced activation, with 32 members showing significant upregulation, particularly in susceptible Newhall orange and Gongkan mandarin and tolerant Guanxi pomelo. In contrast, MYB family genes displayed reduced enrichment in susceptible genotypes (Newhall orange and Gongkan mandarin) compared to other cultivars. Among bHLH TFs, 21 genes demonstrated context-dependent regulation, including *bHLH140* (*Cs_ont_6g014390*), which was strongly induced in four susceptible cultivars but remained unaltered in tolerant genotypes. Notably, two pathogen-triggered immunity (PTI)-associated genes (*Cs_ont_5g018840*, *Cs_ont_2g004240*) were specifically upregulated in Gongkan mandarin and Guanxi pomelo.

### 2.7. Innate Immune Signaling

**Figure 5:**
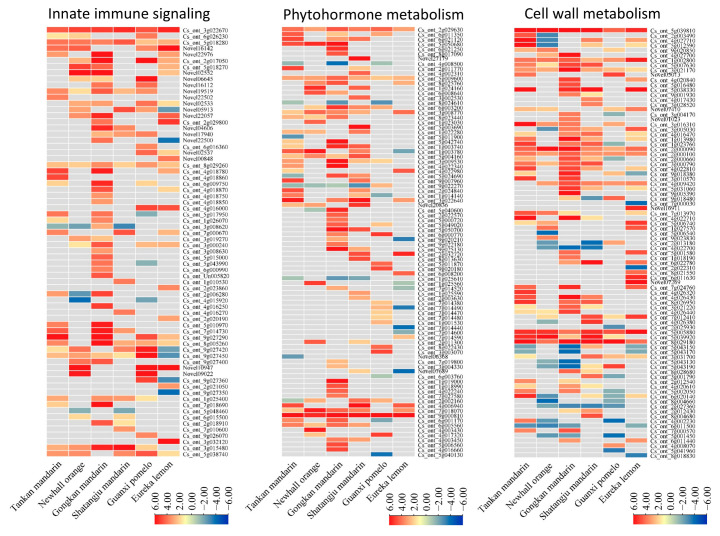
Heatmaps of differentially expressed genes (DEGs) involved in innate immune signaling, phytohormone metabolism, and cell wall metabolism.

A total of 70 DEGs associated with plant innate immune signaling were identified across the six citrus cultivars (Figure 5; Supplementary Table S9). Most DEGs exhibited upregulation, with the highest number observed in the susceptible cultivar Gongkan mandarin. Twenty-two pattern recognition receptor (PRR) genes, including LRR receptor-like serine/threonine-protein kinase *FLS2* and *EFR*, were significantly enriched in Newhall orange, Gongkan mandarin, Guanxi pomelo, and Eureka lemon. Notably, two *FLS2* homologs (*Cs_ont_2g017050*, *Novel06645*) showed marked induction in the Guanxi pomelo. Eight mitogen-activated protein kinase (MAPK) pathway-related DEGs exhibited differential regulation across six cultivars. A *MAPKKK1* gene (*Cs_ont_3g032120*) was exclusively and strongly induced in the Eureka lemon. Two respiratory burst oxidase homolog (RBOH) genes (*Cs_ont_3g015480*, *Cs_ont_5g038740*) were significantly upregulated in all cultivars except the Eureka lemon. Thirty-eight calcium signaling-related DEGs were identified. A total of 8 calcium-dependent protein kinase (CDPK) genes and 11 calmodulin-like (CML) genes with pronounced upregulation were found in the susceptible Gongkan mandarin. A *CDPK8* homolog (*Cs_ont_4g0160005*) was specifically activated in Guangxi pomelo and Eureka lemon. Eleven DEGs were involved in the cyclic nucleotide-gated channel (CNGC), with ten upregulated in four susceptible cultivars and *Cs_ont_9g027350* strongly downregulated in tolerant Eureka lemon.

### 2.8. Phytohormone Metabolism

A total of 88 DEGs associated with phytohormone metabolism were identified across the six citrus cultivars, with distinct activation patterns observed between the susceptible and tolerant genotypes (Figure 5; Supplementary Table S10). Susceptible cultivars demonstrated broader hormonal pathway activation, showing significantly higher DEG enrichment compared to their tolerant counterparts. These DEGs spanned key biosynthesis and signaling pathways for auxin, abscisic acid (ABA), ethylene, salicylic acid (SA), cytokinin, jasmonic acid (JA), and gibberellin (GA).

Auxin-related regulation was particularly prominent in susceptible cultivars (Tankan mandarin, Newhall orange, Gongkan mandarin, and Shatangju mandarin), with 15 DEGs including indole-3-acetate-amide synthetase genes and auxin-responsive proteins (auxin-induced proteins and auxin-binding proteins) predominantly upregulated. The ethylene pathway emerged as the most substantially regulated, with 34 DEGs encompassing 1-aminocyclopropane-1-carboxylate (ACC) synthases and ethylene-responsive transcription factors being significantly enriched in Tankan mandarin, Gongkan mandarin, and Shatangju mandarin.

SA-related regulation exhibited cultivar-specific patterns, with three salicylic acid-binding protein (SABP; *Cs_ont_1g025610*, *Cs_ont_1g025560*, *Cs_ont_1g025590*) genes showing distinct activation. *Cs_ont_1g025560* and *Cs_ont_1g025590* were upregulated in Newhall orange and Shatangju mandarin, respectively, while other SABPs were downregulated. NPR3/4 family members demonstrated divergent expression profiles, including *Cs_ont_7g014520* upregulation in Gongkan mandarin, four homologs (*Cs_ont_7g014380*, *Cs_ont_7g014490*, *Cs_ont_7g014470*, *Cs_ont_7g014480*) activated in Guanxi pomelo, and four members (*Cs_ont_7g014520*, *Cs_ont_7g014490*, *Cs_ont_7g014440*, *Cs_ont_7g014600*) suppressed in Eureka lemon. Additionally, the SA-responsive transcription factor TGA1 (*Cs_ont_1g001530*) was exclusively upregulated in Guanxi pomelo. Cytokinin biosynthesis genes (*Cs_ont_2g011300*, *Cs_ont_8g022430*) were specifically activated in Newhall orange, Shatangju mandarin, and Guanxi pomelo.

JA signaling components, particularly four TIFY transcription factors (*Cs_ont_1g019000*, *Cs_ont_1g018990*, *Cs_ont_4g022240*, *Cs_ont_7g027580*), showed exclusive upregulation in Gongkan mandarin. GA-related DEGs displayed preferential enrichment in susceptible cultivars, with minimal activation observed in tolerant genotypes Guanxi pomelo and Eureka lemon.

### 2.9. Cell Wall Metabolism

A total of 87 DEGs linked to cell wall metabolism were identified across the six citrus cultivars, revealing distinct regulatory patterns between the susceptible and tolerant genotypes (Figure 5; Supplementary Table S11). Cell wall synthesis-related genes were broadly enriched in susceptible cultivars (Tankan mandarin, Gongkan mandarin, Shatangju mandarin, and Newhall orange), though partial downregulation occurred specifically in Newhall orange. Key synthesis components, including *Galactoside 2-alpha-L-fucosyltransferase*, *cellulose synthase A catalytic subunit*, *cellulose synthase-like protein*, *COBRA-like protein*, *Galacturonosyltransferase*, and *UDP-glucuronate–xylan alpha-glucuronosyltransferase*, were upregulated in four susceptible genotypes.

Cell degradation pathways showed contrasting regulation, with 15 pectinesterase-associated DEGs identified. Notably, seven pectinesterase genes (*Cs_ont_7g013970*, *Cs_ont_5g006740*, *Cs_ont_1g027570*, *Cs_ont_5g001580*, *Cs_ont_6g022780*, *Cs_ont_8g021550*, *Cs_ont_6g011630*) were specifically enriched in Eureka lemon. *Nine xyloglucan endotransglucosylase/hydrolase* (*XTH*) genes exhibited predominant upregulation in three susceptible cultivars (Tankan mandarin, Gongkan mandarin, and Shatangju mandarin), indicating active hemicellulose remodeling.

Structural cell wall protein-related DEGs displayed cultivar-specific divergence. DEGs encoding expansins, fasciclin-like arabinogalactan proteins, and proline-rich proteins were predominantly downregulated in Newhall orange, Gongkan mandarin, and Guanxi pomelo, whereas Shatangju mandarin showed a significant upregulation of these structural components. This dichotomy suggested genotype-specific strategies for cell wall reinforcement or flexibility during HLB responses.

## 3. Discussion

Starch accumulation, a hallmark physiological response in HLB-affected plants [16], has been hypothesized to drive leaf mottling and chlorosis through excessive starch deposition disrupting thylakoid integrity or sucrose/glucose accumulation impairing cellular homeostasis [17]. Previous studies have documented the HLB-induced suppression of starch degradation pathways, particularly the downregulation of key catabolic genes (*BAM3*, *MEX1*, *DPE2*) in susceptible *C. sinensis* genotypes during disease progression [18,19]. Our transcriptomic analysis revealed cultivar-specific regulatory patterns in starch metabolism. The β-amylase 3 gene *BAM3* (*Cs_ont_5g043520*), a critical starch degradation enzyme, showed pronounced upregulation exclusively in tolerant cultivars, Guanxi pomelo and Eureka lemon. This enhanced transcriptional activation was contrasted with the suppressed starch catabolism pathways observed in susceptible genotypes, suggesting that elevated *BAM3* expression may bolster starch breakdown capacity in tolerant varieties. Such differential regulation could mitigate chloroplast dysfunction by reducing starch accumulation, thereby attenuating leaf chlorosis in HLB-tolerant cultivars.

Transcriptional profiling revealed divergent defense strategies between HLB-susceptible and -tolerant citrus cultivars, with susceptible genotypes exhibiting a quantitative amplification of TF activation compared to the restrained transcriptional reprogramming observed in tolerant varieties. As master regulators of plant metabolic adjustments, TFs orchestrated both the magnitude and specificity of defense pathway activation [20]. Notably, WRKY family members, key mediators of immune transcriptomes in *Arabidopsis* through MAP kinase-dependent phosphorylation cascades triggered by pathogen-associated molecular patterns [21,22,23], were markedly enriched in susceptible cultivars (Newhall orange, Gongkan mandarin, and Guanxi pomelo), suggesting the hyperactivation of immune signaling pathways. In contrast, MYB TFs, which integrated defense networks and directly regulated flavonoid biosynthesis genes [24,25], showed limited activation in Newhall orange and Gongkan mandarin. This paradoxical divergence in TF engagement (WRKY amplification vs. MYB suppression) underscored unresolved questions about cultivar-specific regulatory mechanisms, particularly regarding the coordination of flavonoid-mediated defenses. A striking genotype-specific pattern emerged with the exclusive upregulation of *bHLH140* (*Cs_ont_6g014390*), a stress-responsive flavonoid regulator [24], in four susceptible cultivars (Figure 4; Supplementary Table S8). Given bHLH TFs’ established role in fine-tuning secondary metabolite production, this susceptibility-linked activation raised compelling questions about whether *bHLH140* induction represents a maladaptive response or failed defense modulation, a hypothesis requiring validation through targeted functional studies.

Comparative transcriptomic profiling revealed distinct immune strategies in citrus cultivars responding to CLas infection, with susceptible genotypes exhibiting hypersensitive signaling and tolerant varieties deploying targeted defense activation. While both groups initiated immune responses, tolerant cultivars demonstrated a refined regulation of key pathways associated with HLB resistance. The primary defense layer, mediated by pattern recognition receptors (PRRs), showed cultivar-specific activation. PRRs contained surface-localized receptor-like kinases (*RLKs*) with ligand-binding extracellular domains and intracellular kinase domains or receptor-like proteins (*RLPs*) lacking signaling domains [26]. Tolerant Guanxi pomelo uniquely upregulated two *FLS2* homologs (*Cs_ont_2g017050*, *Novel06645*) encoding flagellin-sensing receptors, suggesting enhanced PTI activation through the recognition of bacterial epitopes like flg22 and EF-Tu, a mechanism conserved in *Arabidopsis* [27,28]. These RLKs contrasted with the limited PRR activation observed in susceptible cultivars. CLas-induced ROS overproduction, typically mediated by RBOHs [13,29], displayed genotype-specific regulation. Although two RBOH genes (*Cs_ont_3g015480*, *Cs_ont_5g038740*) were upregulated broadly, their subdued expression in tolerant Eureka lemon correlated with restrained ROS accumulation, a potential adaptation to minimize oxidative tissue damage while maintaining defense signaling. Calcium-dependent protein kinases (CDPKs), critical transducers of immune signals [30,31], showed limited enrichment in susceptible cultivars (Newhall orange and Shatangju mandarin), likely contributing to their attenuated defense responses. Strikingly, *CNGC* (*Cs_ont_9g027350*), which mediated Ca^2+^ influx linked to oxidative bursts and pathogen-induced cell death [32,33], was markedly downregulated in Eureka lemon (Figure 5; Supplementary Table S9). This suppression likely stabilized Ca^2+^ homeostasis, reducing necrotic symptom progression, a hallmark of HLB tolerance.

Phytohormones served as central regulators of plant development and defense against pathogen infections [34]. Comparative analysis revealed the heightened activation of hormone-related pathways in susceptible cultivars, with tolerant varieties exhibiting fewer enriched genes in these pathways. Susceptible genotypes, including Tankan mandarin, Newhall orange, and Shatangju mandarin, predominantly upregulated ethylene biosynthesis and signaling genes in response to CLas infection, while Gongkan mandarin activated both ethylene and jasmonic acid pathways. In contrast, the tolerant cultivar Eureka lemon triggered SA-mediated defense transcription, whereas Guanxi pomelo showed no distinct hormonal enrichment. Ethylene-related genes were markedly upregulated in three susceptible cultivars (Tankan mandarin, Gongkan mandarin, Shatangju mandarin). Pathogen-induced ethylene accumulation often exacerbates disease progression by accelerating senescence [35,36], consistent with prior observations in CLas-infected sweet orange [18]. This suggested that ethylene hyperactivation in susceptible genotypes may facilitate HLB symptom development. SA, a critical defense hormone, transduced extracellular *PAMP* signals to activate defense gene transcription [37]. Notably, multiple *NPR3* genes, *NPR4* genes, and SA receptors that negatively regulated defense responses were downregulated in Eureka lemon. SA potentiates immunity by suppressing *NPR3* and *NPR4* activity, thereby derepressing downstream defense regulators [38]. This regulatory mechanism likely underpinned Eureka lemon’s SA pathway activation and subsequent defense gene induction.

The plant cell wall, primarily composed of polysaccharides (cellulose, hemicellulose, and pectin), serves as a critical physical barrier against pathogen invasion [39]. The key genes involved in wall reinforcement, *cellulose synthase A catalytic subunit*, *cellulose synthase-like protein*, and *COBRA-like protein*, were markedly enriched in four susceptible cultivars (Tankan mandarin, Gongkan mandarin, Shatangju mandarin, and Newhall orange), suggesting an enhanced synthesis of structural polysaccharides as a compensatory defense mechanism [40,41]. Pectin, the predominant polysaccharide in dicot cell walls, requires precise regulation by pectin methylesterases (PMEs) and their inhibitors (PMEIs) to maintain cell wall integrity. PME-mediated de-esterification of homogalacturonan was essential for pectin crosslinking and wall rigidity, a process implicated in antibacterial and antiviral defenses [42,43]. Notably, Eureka lemon exhibited a pronounced enrichment in PME-encoding genes compared to other cultivars, potentially stabilizing cell wall architecture to impede pathogen penetration. Conversely, genes encoding xyloglucan endotransglucosylase/hydrolase (XTH) proteins, enzymes associated with wall loosening, were upregulated in susceptible cultivars (Tankan mandarin, Gongkan mandarin, Shatangju mandarin) (Figure 5; Supplementary Table S11). As XTH activity facilitated cell wall remodeling during pathogen invasion [41], this dysregulation may compromise structural defenses, creating entry points for CLas colonization.

Our identification of defense-related pathways and key regulators in HLB-affected citrus aligned with strategies used against other major vascular diseases, such as mal secco disease (caused by *Plenodomus tracheiphilus*) in lemon across the Mediterranean basin and the Black Sea region [44,45]. Similarly to how mal secco management in citrus relies on deploying tolerant rootstock–scion combinations to restrict xylem colonization [46,47], our identification of cell wall reinforcement and salicylic acid-mediated signaling pathways provided clear molecular targets for breeding HLB-tolerant cultivars. Integrating these candidate mechanisms into marker-assisted selection will accelerate the translation of transcriptomic discoveries into disease-resilient varieties.

Although our transcriptomic analyses had identified candidate host genes involved in HLB tolerance, functional validation, such as CRISPR/Cas9 knock-out or transgenic overexpression in citrus, will be required to establish causal roles. Additionally, future studies should include additional genotypes within each tolerance category and assess variability across multiple tolerant and susceptible cultivars to distinguish generalizable tolerance mechanisms from cultivar-specific effects. This study focused on global transcriptomic contrasts between tolerant and susceptible groups and did not correlate gene expression with detailed physiological or disease severity phenotypes. Addressing these points in follow-up experiments, such as longitudinal sampling, targeted functional assays, and integrated phenotyping, will be essential to translate our findings into breeding strategies for HLB tolerance.

## 4. Materials and Methods

### 4.1. Plant Material

Six commercially important citrus cultivars were collected from major citrus-producing regions in South China, including the following: Gongkan mandarin (*Citrus reticulata* cv. Gongkan, Zhaoqing, Guangdong Province), Tankan mandarin (*C. reticulata* cv. Tankan, Chaozhou, Guangdong Province), Shatangju mandarin (*C. reticulata* Blanco cv. Shatangju, Huizhou, Guangdong Province), Eureka lemon (*C. limon* cv Eureka, Heyuan, Guangdong Province), Guanxi pomelo (*C. maxima* cv Guanxi Yu, Meizhou, Guangdong Province), and Newhall orange (*C. sinensis* Osbeck cv. Newhall, Ganzhou, Jiangxi Province). According to a previous study [6], *C. limon* and *C. maxima* were more tolerant to HLB compared to *C. reticulata* Blanco and *C. sinensis*. All leaf samples were taken from the fully expanded leaves of the current-season vegetative flush. For each cultivar, symptomatic leaves exhibiting HLB-associated chlorosis/mottling and asymptomatic leaves from healthy trees were flash-frozen in liquid nitrogen and stored at −80 °C. Three biological replicates per cultivar (healthy vs. infected) were processed for RNA-Seq.

### 4.2. DNA and RNA Extraction

For DNA extraction, fresh leaf midrib tissues (100 mg) were homogenized using an MP FastPrep^®^-24 grinder (MP Biomedicals, Irvine, CA, USA) at 4 m/s for 1 min. Total DNA was isolated using the EZNA HP Plant DNA Kit (Omega Bio-Tek, Norcross, GA, USA). For RNA isolation, 250 mg pooled midrib tissues from three leaves per biological replicate was processed with the EZNA Total RNA Kit I (Omega Bio-Tek, Norcross, GA, USA). Nucleic acid yields were quantified via a Qubit 2.0 Fluorometer (Thermo Fisher Scientific, Waltham, MA, USA). RNA integrity was validated by capillary electrophoresis (Agilent 2100 Bioanalyzer, Agilent Technologies, Santa Clara, CA, USA).

### 4.3. RNA Sequencing and Transcriptomic Analysis cDNA Library Production

All cDNA libraries were prepared using the NEBNext^®^ Ultra™ RNA Library Prep Kit (Illumina-compatible; New England Biolabs, Ipswich, MA, USA) and sequenced on an Illumina HiSeq 3000 platform (150 bp paired-end reads; Novogene, Beijing, China). Raw reads were filtered for quality and aligned to the Citrus sinensis reference genome (GCA_022201045.1) [48] via Tophat2 with ≤2 mismatches [49]. Gene expression quantification was performed using HTSeq v0.61, with normalized RPKM values calculated for cross-sample comparisons. Differential expressed genes (DEGs) between CLas-infected and control groups was identified by DEGseq with the cut-off values set as |log2(fold change)| ≥ 1.5 and q < 0.005 [50]. The functional annotation of significant DEGs included Gene Ontology (GO) enrichment via GOseq (FDR < 0.05) [51] and KEGG pathway analysis using KOBAS 2.0 [52].

### 4.4. CLas Quantification

CLas pathogen load was determined through TaqMan qPCR using the CLas4G/HLBr primer–probe system (CLas4G: AGTCGAGCGCGTATGCGAAT; HLBr: GCGTTATCCCGTAGAAAAAGGTAG; HLBp: FAM-AGACGGGTGAGTAACGCG-BHQ) established in prior work [53]. Reactions (20 μL) contained 10 μL PerfectStart^®^ II Probe qPCR SuperMix, 1 μL genomic DNA (25 ng), 0.4 μL of each primer (10 μM), 0.2 μL probe (10 μM), and 8 μL ddH_2_O. All PCRs were performed on a Bio-Rad CFX Connect system. Thermal cycling involved initial denaturation (95 °C, 2 min), followed by 40 cycles of 95 °C for 10 s and 58 °C for 30 s (fluorescence acquisition). Amplification data were analyzed via CFX Manager 2.1 software with automated baseline/threshold determination.

### 4.5. Gene Expression Validation

Ten DEGs spanning functional categories including cell wall metabolism (*Cs_ont_5g039810*, *Cs_ont_2g000090*), secondary metabolites (*Cs_ont_6g005240*), transcription factors (*Cs_ont_6g014390*, *Cs_ont_5g018840*), innate immune signaling (*Cs_ont_2g017050*, *Cs_ont_3g015480*, *Cs_ont_9g027350*), phytohormone metabolism (*Cs_ont_9g000810*), and starch and sucrose metabolism (*Cs_ont_5g002310*) were selected for RT-qPCR validation. All primer sets are listed in Supplementary Table S12. Utilizing the original RNA samples from transcriptome profiling, cDNA synthesis was performed with TransScript^®^ One-Step gDNA Removal and cDNA Synthesis SuperMix (TransGen Biotech, Beijing, China). qPCRs (20 μL) employed TransStart^®^ Green qPCR SuperMix (TransGen Biotech), 1 μL cDNA, 0.4 μL gene-specific primers (10 μM; Supplementary Table S12), and 8 μL ddH_2_O, processed on a Bio-Rad CFX Connect system under standardized conditions: 95 °C/30 s initiation; 40 cycles of 95 °C/10 s; and 60 °C/30 s (fluorescence acquisition at 60 °C). Normalized expression (*GAPDH* reference) was calculated via the 2^−ΔΔCt^ method, with CLas-infected/mock-control fold changes (Log2) compared to the RNA-Seq results. The expression profiles of ten selected genes generated by RT-qPCR were consistent with RNA-Seq data, indicating the reliability of RNA-Seq analysis in this study (Supplementary Figure S1).

## 5. Conclusions

This study provides a comprehensive transcriptomic analysis of six major citrus cultivars (four susceptible, two tolerant) in South China, revealing genotype-specific responses to the HLB RNA sequencing of symptomatic and asymptomatic leaves uncovered via extensive transcriptional reprogramming, with DEGs involved in starch and sucrose metabolism, secondary metabolism, transcriptional regulation, innate immune signaling, phytohormone pathways, and cell wall metabolism. Notably, tolerant cultivars (*C. limon* and *C. maxima*) exhibited an enhanced activation of starch degradation genes, such as β-amylase 3, which may mitigate chloroplast dysfunction by reducing excessive starch accumulation. In contrast, susceptible cultivars (*C. reticulata* and *C. sinensis*) displayed a hyperactivation of defense-related pathways, particularly ethylene biosynthesis and WRKY transcription factors, potentially exacerbating disease symptoms. The differential regulation of pattern recognition receptors, calcium signaling components, and cell wall-modifying enzymes further underscored the distinct defense strategies employed by tolerant versus susceptible genotypes. These insights lay a foundation for identifying molecular markers and targets for breeding HLB-tolerant varieties. By mapping time-course expression profiles, performing gene-level manipulations, and linking transcriptomic changes to physiological outcomes, future studies will build upon this work to accelerate the development of robust, HLB-resistant citrus cultivars.

## Data Availability

All sequence reads generated in this project are available in the NCBI Short Read Archive under BioProject PRJNA990698.

## Supplementary Materials

The following supporting information can be downloaded at https://www.mdpi.com/article/10.3390/ijms26157359/s1.
