# Supplementary material for: Transcriptomic Reprogramming and Key Molecular Pathways Underlying Huanglongbing Tolerance and Susceptibility in Six Citrus Cultivars
Source: Int J Mol Sci. 2025 Jul 30;26(15):7359. doi: 10.3390/ijms26157359 (PMC12347598; doi:10.3390/ijms26157359)
Supplement: Supplementary file 1 [file ijms-26-07359-s001.zip › Supplementary Figure S1 7.12.pdf]

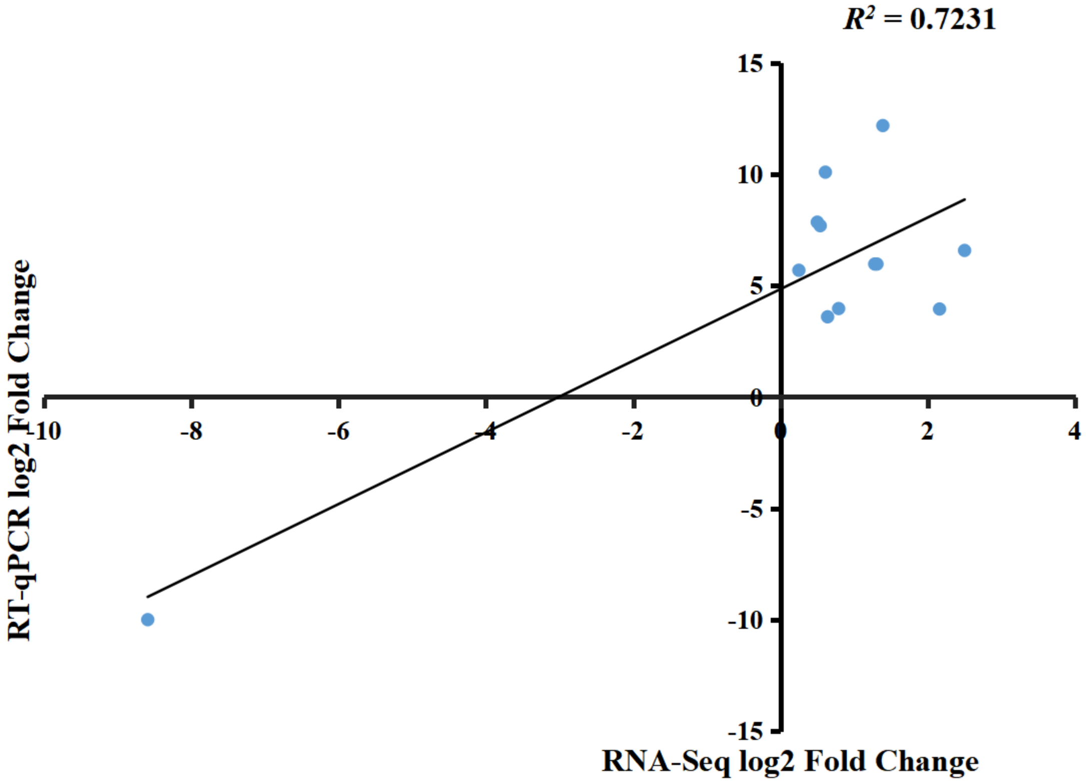

Supplementary Figure S1. Correlation of 10 selected DEGs gene expression between RNA-seq and qPCR validation in six cultivars.
